# The impact of sitting time and physical activity on major depressive disorder in South Korean adults: a cross-sectional study

**DOI:** 10.1186/s12888-017-1439-3

**Published:** 2017-07-28

**Authors:** Jin Young Nam, Juyeong Kim, Kyoung Hee Cho, Jaewoo Choi, Jaeyong Shin, Eun-Cheol Park

**Affiliations:** 1 Department of Public Health, Graduate School, Yonsei University, Seoul, Republic of Korea; 2 Institute of Health Services Research, Yonsei University, Seoul, Republic of Korea; 3 Busan Public Health Policy Institute, Busan, Republic of Korea; 4 Department of Preventive Medicine, Yonsei University College of Medicine, 50 Yonsei-ro, Seodaemun-gu, 120-752 Seoul, Republic of Korea

**Keywords:** Major depressive disorder, PHQ-9, Physical activity, Sedentary behavior, Sitting time

## Abstract

**Background:**

Previous studies have examined associations between sitting time and negative health outcomes and mental health. However, the relationship between overall sitting time and major depressive disorder (MDD) in South Korea has not been studied. This study examined the association between MDD and overall sitting time and physical activity in South Koreans.

**Methods:**

Data from the sixth Korean National Health and Nutrition Examination Survey (KNHANES), a cross-sectional, nationally representative survey, were analyzed. Total participants were 4145 in 2014. MDD was assessed using the Patient Health Questionnaire (PHQ-9). Participants’ data regarding self-reported sitting time and physical activity were analyzed via multiple logistic regression.

**Results:**

Results showed that people who sat for 8–10 h (OR: 1.56, 95% CI: 1.15–2.11) or more than 10 h (OR: 1.71, 95% CI: 1.23–2.39) had increased risk of MDD compared to those who sat for less than 5 h a day. Subgroup analysis showed that the strongest effect of reported sitting time on risk of MDD was found in men with lower levels of physical activity who sat for 8 to 10 h (OR: 3.04, 95% CI: 1.15–8.01) or more than 10 h (OR: 3.43, 95% CI: 1.26–9.35). Level of physical activity was not an independent predictor for MDD.

**Conclusions:**

Sitting for long periods was associated with greater risk of MDD in South Korean adults. Reducing sitting time in people with MDD could help to prevent associated physical health problems and may improve mental health.

**Electronic supplementary material:**

The online version of this article (doi:10.1186/s12888-017-1439-3) contains supplementary material, which is available to authorized users.

## Background

Major depressive disorder (MDD) is an important public health issue, with a lifetime prevalence of approximately 11–15% worldwide [1]. The World Health Organization (WHO) recently stated that MDD was expected to be the second leading cause of disability and fourth leading contributor to the global burden of disease by 2020 [2]. Moreover, MDD not only contributes to the overall global burden of disease but also leads to suicide in some individuals [3]. In fact, South Korea has the highest suicide rate of those reported by Organization for Economic Co-operation and Development (OECD) countries. The lifetime prevalence of MDD based on DSM-IV was 7.5% [4] while other research presented the prevalence of MDD based on CESD-11 as 11 and 10.6%, respectively [5, 6], which were not high rates of MDD compared to those of OECD countries, and the lowest level of antidepressant consumption in Korea has been recorded as the lowest of these countries [7]. The relatively fewer expressions for depressed mood in Asian populations may have resulted in under-reporting of depressive disorders [8]. These rates could be considered a proxy indicator of the population’s mental health. [9]. To reduce this burden, it is necessary to identify crucial risk factors associated with MDD prevention and management.

Physical activity (PA) is known to be associated with a decreased risk of mortality and morbidity resulting from chronic conditions such as cardiovascular disease (CVD) [10, 11], type 2 diabetes [12–14], obesity [15, 16], and cancer [17–19]. Moreover, recent research suggests that PA is related to mental health outcomes [20, 21]. Several recent studies have identified sedentary behavior (SB) as another lifestyle factor associated with poor cardiovascular [22–24] and metabolic health outcomes [25, 26] and reduced life expectancy [27, 28]. Recently, numerous studies have reported on the relationship between sitting time, PA, and MDD [29], as well as on the effects of interaction between sedentary time and PA which may affect mental well-being and productivity [30], or a specifically, divided PA (domain of PA or Social context of PA) and/or SB (time spent on sitting while watching TV or using computer, sitting for travel or overall sitting time, or sitting around throughout the week) [21, 31] were related to mental health outcomes; however, such effects are still less well-known.

Numerous studies have reported relationships between SB and mental health in older adults [32, 33], disadvantaged women [21], middle-aged women [29], employed adults [31], and patients with schizophrenia [26]; however, the definition of SB included only leisure-time, such as that spent watching TV and/or using a computer or the Internet [34, 35]. Moreover, no studies have examined associations between MDD and PA and overall sitting time including that involved in work, leisure, home-based activities, and transportation. Therefore, the aims of this study were to examine the relationship between sitting-time and MDD and estimate the effects of sitting-time and PA on MDD in a representative South Korean population.

## Methods

### Participants, design and setting of study

The study used data collected during the second year (2014) of the sixth Korean National Health and Nutrition Examination Survey (KNHANES), which was conducted by the Korea Centers for Disease Control and Prevention (KCDC) in South Korea. The KNHANES is a cross-sectional, nationally representative survey that has been conducted regularly since 1998, to examine the general health and nutritional status of Korean citizens. The initial sample included 9701 individuals aged >1 year, who were invited to participate in the second year (2014) of the sixth KNHANES. Of these, 7550 completed the survey (response rate: 77.8%).

The study included adults aged ≥20 years who had participated in the 2014 survey. Data for individuals who were younger than 19 years of age (*n* = 1653) or had been diagnosed with cancer (*n* = 114) and those for whom no information regarding depressive symptoms, sitting times, or covariates was provided (*n* = 710) were excluded from the study. The total number of eligible participants was 4145 (1664 men, 2481 women).

### Measures

#### MDD: Patient health questionnaire (PHQ-9)

MDD was assessed using the Patient Health Questionnaire (PHQ-9), which incorporated the 9 criteria upon which a diagnosis of depressive disorder is based in the Diagnostic and Statistical Manual of Mental Disorders, Fourth Edition [36]. Participants were asked to indicate how often each depressive symptom had occurred during the preceding 2 weeks by choosing one of the following options 0 (*not at all*), 1 (*several days*), 2 (*more than half of the days*), and 3 (*nearly every day*) [36]. Total possible scores range from 0 to 27, and cut-off points of 5, 10, 15, and 20 are usually used as thresholds for mild, moderate, moderately severe, and severe depression, respectively, with a score of 10 recommended as the cut-off point for MDD [36]. However, a previous systematic review of 18 validation studies reported that PHQ-9 cut-off scores between 8 and 11 had acceptable diagnostic characteristics for the detection of MDD [37]. Therefore, a score of 8 was used as the cut-off point for MDD in the current study.

#### Overall daily sitting time: International physical activity questionnaire (IPAQ)

Overall daily sitting time was estimated using the long-version of the IPAQ [38, 39] and assessed via the following question: *How many hours do you typically spend sitting or lying down while engaged in activities such as working at a desk or computer, visiting friends, driving, reading, writing, watching TV, playing games, using the Internet, or listening to music on a usual day?* Responses were divided into 4 categories using quartiles: *<5 h/d, 5–7 h/d, 8–10 h/d,* and *>10 h/d.*

#### Physical activity (PA): International physical activity questionnaire (IPAQ)

PA was assessed using the Korean version of the long-format IPAQ [39] and estimated using aerobic activity recommended by the Centers for Disease Control and Prevention and the American College of Sports Medicine [40]. Responses were divided into 2 categories; participants who engaged in at least 150 min of moderate-intensity PA per week, at least 75 min of vigorous-intensity aerobic activity per week, or a combination of moderate- and vigorous-intensity PA (e.g., 1 min of vigorous-intensity PA and 2 min of moderate-intensity PA) for at least 75 min per week were classed as engaging in PA, and those who did not were classed as not engaging in PA.

### Covariates

Age, household income, educational level, occupation, and marital status were included as socioeconomic factors. Health-related factors included obesity, current smoking status, frequency of alcohol use, and number of chronic diseases. Obesity was assessed according to body mass index, which was divided into four groups: underweight (<18.5 kg/m^2^), moderate (18.5–24.9 kg/m^2^), overweight (25.0–29.9 kg/m^2),^ and obese (≥30 kg/m^2^). Current smoking status was a dichotomous variable, as follows: current smokers or those who had smoked more than 100 cigarettes throughout their lives, and those who had never smoked or had previously smoked less than 100 cigarettes throughout their lives. Frequency of alcohol use was calculated according to the average frequency (more than once per month or never) with which alcohol was consumed during the preceding year. Number of chronic diseases was classified into 3 categories according to the number of diseases, including high blood pressure, dyslipidemia, stroke, myocardial infarction, angina, arthritis, rheumatoid arthritis, chronic renal failure, asthma, thyroid disease, and hepatitis B, reported (0, 1, or ≥2).

### Statistical analysis

General characteristics were evaluated using chi-square tests. Multiple logistic regression models were created to determine whether sitting time was related to depressive symptoms. In addition, Cochran-Armitage trend test was used to investigate whether there was a linear trend in the relationship between sitting time and MDD. In addition, subgroup analysis was performed according to PA, occupation, and sitting time, using multiple logistic regression. All statistical analyses were performed using SAS 9.4 (SAS Institute, Inc., Cary, NC).

## Results

Table 1 shows the participants’ general characteristics. The 4145 participants included 1664 men and 2481 women; of these, 112 (6.7%) men and 312 (12.6%) women reported MDD. Sitting time of ≥8 h/d was reported by 45.6% of participants. Specifically, men tended to sit excessively more than women. The proportion of men (20.01%) who sat for >10 h/d was higher compared to women (16.36%; Additional file 1: Table S1).

**Table 1:** Participants’ general characteristics

	Depression						
	Total		Yes		No		
	N	(%)	N	(%)	N	(%)	*p*-value
Sitting time (hours)							
< 5	1059	(25.55)	86	(8.12)	973	(91.88)	0.0012
5–7	1195	(28.83)	107	(8.95)	1088	(91.05)	
8–10	1152	(27.79)	136	(11.81)	1016	(88.19)	
>10	739	(17.83)	95	(12.86)	644	(87.14)	
Sex							
Men	1664	(40.14)	112	(6.73)	1552	(93.27)	<.0001
Women	2481	(59.86)	312	(12.58)	2169	(87.42)	
Age (year)							
20–29	422	(10.18)	53	(12.56)	369	(87.44)	0.0056
30–39	753	(18.17)	69	(9.16)	684	(90.84)	
40–49	728	(17.56)	56	(7.69)	672	(92.31)	
50–59	806	(19.45)	77	(9.55)	729	(90.45)	
60–69	758	(18.29)	78	(10.29)	680	(89.71)	
70+	678	(16.36)	91	(13.42)	587	(86.58)	
Household income level							
Low	759	(18.31)	157	(20.69)	602	(79.31)	<.0001
Lower middle	1030	(24.85)	105	(10.19)	925	(89.81)	
Upper middle	1214	(29.29)	88	(7.25)	1126	(92.75)	
High	1142	(27.55)	74	(6.48)	1068	(93.52)	
Educational levels							
Middle school or below	1439	(34.72)	196	(13.62)	1243	(86.38)	<.0001
High school	1332	(32.14)	128	(9.61)	1204	(90.39)	
Above college	1374	(33.15)	100	(7.28)	1274	(92.72)	
Occupation							
White collar	927	(22.36)	50	(5.39)	877	(94.61)	<.0001
Pink collar	500	(12.06)	53	(10.60)	447	(89.40)	
Blue collar	989	(23.86)	76	(7.68)	913	(92.32)	
Unemployed, housewife or students	1729	(41.71)	245	(14.17)	1484	(85.83)	
Marital status							
Single	564	(13.61)	66	(11.70)	498	(88.30)	<.0001
Married	3069	(74.04)	259	(8.44)	2810	(91.56)	
Separated	512	(12.35)	99	(19.34)	413	(80.66)	
Physical activity							
No	1968	(47.48)	228	(11.59)	1740	(88.41)	<.0001
Yes	2177	(52.52)	196	(9.00)	1981	(91.00)	
Obesity (BMI, kg/m^2^)							
Low-weight (< 18.5)	171	(4.13)	33	(19.30)	138	(80.70)	<.0001
Normal (18.5–24.9)	2652	(63.98)	258	(9.73)	2394	(90.27)	
Overweight (25.0–29.9)	1149	(27.72)	107	(9.31)	1042	(90.69)	
Obesity (≥ 30.0)	173	(4.17)	26	(15.03)	147	(84.97)	
Current Smoking status							
No	3408	(82.22)	340	(9.98)	3068	(90.02)	<.0001
Yes	737	(17.78)	84	(11.40)	653	(88.60)	
Alcohol use							
No	1986	(47.91)	226	(11.38)	1760	(88.62)	<.0001
Yes	2159	(52.09)	198	(9.17)	1961	(90.83)	
Number of chronic diseases^a^							
0	2239	(54.02)	196	(8.75)	2043	(91.25)	<.0001
1	1133	(27.33)	110	(9.71)	1023	(90.29)	
≥ 2	773	(18.65)	118	(15.27)	655	(84.73)	
Total	4145	(100.00)	424	(10.23)	3721	(89.77)	

^a^Number of chronic diseases: Hypertension, dyslipidemia, stroke, myocardial infarction, angina, arthritis, rheumatoid arthritis, asthma, thyroid gland disorder, chronic renal failure, hepatitis B

Table 2 presents the estimated odds ratios (ORs) from the multiple logistic regression analysis. Risk of MDD in women was twice that observed in men (OR: 2.00, 95% CI: 1.49–2.68). Regarding sitting time, risk of MDD in men who sat for >10 h/d was greater relative to those who sat for <5 h/d (OR: 2.04, 95% CI: 1.12–373). Similarly, risk of MDD in women who sat for ≥8 h/d was greater relative to that of those who sat for <5 h/d (8–10 h/d: OR: 1.59, 95% CI: 1.12–2.27; >10 h/d: OR: 1.64, 95% CI: 1.09–2.45). There was a significant association between linear trends of sitting time and MDD (*P* = 0.0001). Both men and women who sat for longer periods were significantly more likely to report higher MDD compared to those who sat for <5 h/d (*P* = 0.0053 and *P* = 0.0040, respectively). Low level of physical activity was not an independent risk factor for MDD in men or women.

**Table 2:** Association between sitting time and major depressive disorder

	Total		Men		Women	
	OR	95% CI	OR	95% CI	OR	95% CI
Sitting time (hours)						
< 5	1.00		1.00		1.00	
5–7	1.09	(0.80-1.48)	0.83	(0.45-1.53)	1.18	(0.82-1.69)
8–10	1.56	(1.15-2.11)	1.59	(0.89-2.84)	1.58	(1.10-2.25)
> 10	1.71	(1.23-2.39)	2.04	(1.12-3.73)	1.62	(1.08-2.42)
P for trend^b^	0.0001		0.0053		0.004	
Sex						
Male	1.00					
Female	1.99	(1.49-2.67)				
Age (year)						
20–29	2.66	(1.40-5.02)	1.97	(0.62-6.29)	2.82	(1.31-6.06)
30–39	2.55	(1.54-4.23)	4.27	(1.72-10.58)	1.75	(0.93-3.31)
40–49	1.85	(1.14-3.02)	2.50	(1.02-6.10)	1.43	(0.78-2.62)
50–59	1.80	(1.21-2.67)	1.86	(0.85-4.06)	1.65	(1.03-2.65)
60–69	1.27	(0.89-1.80)	0.83	(0.40-1.71)	1.39	(0.92-2.10)
70+	1.00		1.00		1.00	
Household income level						
Low	3.15	(2.21-4.50)	4.31	(2.14-8.69)	3.00	(1.97-4.57)
Lower middle	1.42	(1.02-1.98)	1.42	(0.76-2.68)	1.49	(1.00-2.20)
Upper middle	1.03	(0.74-1.44)	0.80	(0.43-1.50)	1.15	(0.78-1.70)
High	1.00		1.00		1.00	
Educational levels						
Middle school or below	1.41	(0.96-2.07)	1.44	(0.72-2.89)	1.30	(0.81-2.08)
High school	1.17	(0.86-1.59)	1.27	(0.72-2.25)	1.15	(0.80-1.67)
Above college	1.00		1.00		1.00	
Occupation						
White collar	1.00		1.00		1.00	
Pink collar	1.80	(1.16-2.81)	3.24	(1.50-7.01)	1.40	(0.81-2.40)
Blue collar	1.26	(0.83-1.93)	1.57	(0.77-3.19)	1.12	(0.65-1.94)
Unemployed, housewife or students	2.07	(1.44-2.98)	2.47	(1.16-5.26)	1.86	(1.20-2.88)
Marital status						
Single	1.13	(0.74-1.74)	1.45	(0.74-2.84)	0.85	(0.48-1.51)
Married	1.00		1.00		1.00	
Separated	1.46	(1.09-1.96)	1.56	(0.76-3.19)	1.44	(1.03-2.02)
Physical activity						
No	1.13	(0.91-1.41)	1.04	(0.69-1.58)	1.16	(0.90-1.50)
Yes	1.00		1.00		1.00	
Obesity (BMI, kg/m^2^)						
Low-weight (< 18.5)	2.01	(1.31-3.09)	3.72	(1.61-8.62)	1.79	(1.08-2.97)
Normal (18.5–24.9)	1.00		1.00		1.00	
Overweight (25.0–29.9)	0.87	(0.67-1.11)	1.15	(0.73-1.81)	0.74	(0.54-1.01)
Obesity (≥ 30.0)	1.42	(0.89-2.26)	1.13	(0.41-3.11)	1.50	(0.89-2.55)
Current Smoking status						
No	1.00		1.00		1.00	
Yes	1.94	(1.41-2.66)	2.05	(1.34-3.14)	1.94	(1.16-3.25)
Alcohol use						
No	1.00		1.00		1.00	
Yes	1.09	(0.87-1.38)	1.21	(0.76-1.95)	1.11	(0.85-1.45)
Number of chronic diseases^a^						
0	1.00		1.00		1.00	
1	1.17	(0.88-1.57)	1.11	(0.66-1.87)	1.13	(0.79-1.61)
≥ 2	1.67	(1.20-2.33)	1.79	(0.95-3.36)	1.55	(1.05-2.30)

^a^Number of chronic diseases: Hypertension, dyslipidemia, stroke, myocardial infarction, angina, arthritis, rheumatoid arthritis, asthma, thyroid gland disorder, chronic renal failure, hepatitis B

^b^Cochran-armitage trend test

**Fig. 1:**
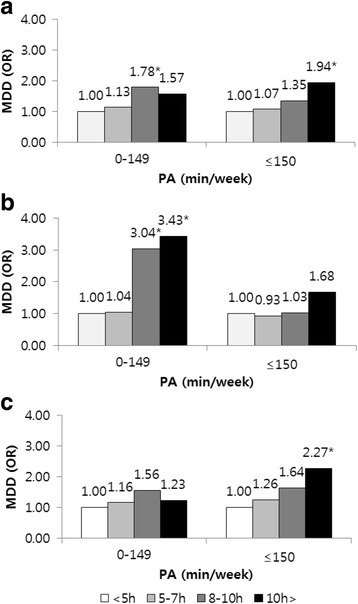
Subgroup analysis of sitting-time and major depressive disorder according to physical activity. **a** Differences in MDD according to sitting-time and PA in total participants. **b** Differences in MDD according to sitting-time and PA in men. **c** Differences in MDD according to sitting-time and PA in women. Adjusted for age, household income level, educational level, marital status, occupation, obesity, current smoking status, alcohol use and number of chronic diseases. * *P* < 0.05, ** *p* < 0.01, *** *p* < 0.001. † MDD: Major depressive disorders, PA: Physical activity

Figure 1 depicts the subgroup analysis in which multiple logistic regression was performed to assess the relationship between sitting time and MDD according to PA. Results showed that men who sat for ≥8 h/d were at significantly high risk of MDD compared to those who sat for <5 h/d (8–10 h/d: OR: 3.04, 95% CI: 1.15–8.01; >10 h/d: OR: 3.43, 95% CI: 1.26–9.35). Women who sat for >10 h/d were at greater risk of MDD compared to those who sat for <5 h/d (OR: 2.27, 95% CI: 1.23–4.21) (Additional file 1: Table S2).

## Discussion

This study is the first to consider evidence for the effect of overall sitting time on risk of MDD in Korea. Using cross-sectional design, our study found that sitting time was positively associated with MDD. Sitting for long periods (>10 h/d) was significantly related to higher risk of MDD in both men and women. Sitting for 8–10 h/d was associated with risk of MDD in both sexes; however, the association was statistically significant only in women. In addition, PA was inversely related to MDD risk, although this association was nonsignificant.

The results are consistent with those of previous studies indicating that longer periods of sitting are related to poor mental health [21, 29, 32, 33, 41] and PA was negatively associated with risk of depression [20, 21]. However, these studies did not examine sex differences in health outcomes related to SB; one study involved both men and women, but did not examine potential interactions according to sex [33], while others involved only women or assessed the relationship between leisure-related sitting time, such that spent watching TV or using a computer, and depressive symptoms [21, 35]. Moreover, a sex difference has previously been observed in mental health outcomes related to sitting time, with findings partially similar to the current results. In the current study, sitting for >7 h/d was associated with greater MDD risk in women relative to that observed in men [31]; however, MDD risk in men who sat for >10 h/d was twice that of men who sat for <5 h/d and higher relative to that of women.

The findings of the current study indicated that there was no interaction between sitting time, PA, and MDD risk; this may have occurred because of the small sample size. However, subgroup analysis was performed because PA is an important factor in the attenuation of depression [29]. Interestingly, the results showed a sex difference. MDD risk in men who did not engage in PA and sat for ≥8 h/d was approximately 3 times higher relative to that of those who did not engage in PA and sat for <5 h/d. In women, MDD risk in those who did not engage in PA and sat for ≥8 h/d was higher relative to that of those who sat for <5 h/d, but the associations between MDD and sitting time and PA were nonsignificant. This indicates that men who did not engage in PA and sat for long periods were likely to experience MDD; Interestingly, risk of MDD in women who engaged in PA and sat for >10 h/d was more than twice that of those who sat for <5 h/d. This could have occurred because half of the women in the study population, such as housewives who engaged in PA via daily chores, were unemployed, and unemployed people were at greater risk of MDD relative to employed individuals (Table 2); therefore, MDD risk in women who engaged in PA could have differed according to sitting time.

The results of the current study are consistent with those of a previous Spanish study [35] that examined the combined effect of sitting time and PA on depression. The results showed that risk of mental disorders in those with high PA levels and short periods of SB was 25% lower relative to that of those with low PA levels and long periods of SB. Moreover, Lucas et al. examined the combined effect of PA and time spent watching TV on depression. Their results suggested that both factors contribute to depression risk, because watching TV typically replaces PA [34]. These studies identified SB as an important correlate of decreased MDD risk when PA levels were low but not when they were high, suggesting that PA could affect the relationship between SB and depression. Even though this was a cross-sectional study and did not explain causal relationships, sitting for long periods and failure to engage in PA increased MDD risk, which is consistent with the findings of previous studies.

Some studies have described potential mechanisms underlying the inverse relationships between PA and sitting-time and MDD. One possible explanation for the positive relationship between sitting time and MDD risk is that sitting time might replace PA [20, 34]. A few studies have suggested that the more time adults spend sitting during activities, such as watching TV, the less time they spend engaging in PA [42]. Numerous studies have shown that PA is not only associated with a reduced risk of negative health outcomes [10–17] but also involved physiological mechanisms including changes in endorphins, core body temperature, central serotonergic systems, and brain activation involved in emotional regulation [43–45]. Another possible explanation for the association between sitting for long periods and high risk of depression involves the social withdrawal hypothesis. Psychosocial mechanisms include distraction, enhanced feelings of control and mastery, improved self-esteem and self-efficacy, behavioral activation, and social interaction [46–48]. For instance, as the frequency with which people watch TV or use a computer or the Internet increases, they become further removed from social interaction, which increases their risk of depression [49]. However, this type of activity must be contrasted with computer or Internet use for work or communication [50, 51]. Nevertheless, physiological or psychological mechanisms could support the relationship between sitting time and MDD risk. South Korea has the highest proportion of households with broadband Internet access (97.2%) [52] and the highest business use of broadband (98.4% of businesses with 10 or more employees) [52] of the OECD countries, along with high levels of mobile penetration and daily mobile use for long periods [53]. It is easy to be exposed to the Internet via computers or mobile phones in South Korea, which could affect the relationships between MDD and sitting-time and PA. As a result, people with depression might have poorer physical health because they were more likely to choose a sedentary lifestyle due to their depressed feeling, fatigue, or evasion of social interaction.

### Limitations

The study was subject to several limitations. First, it was a cross-sectional study and could not explain whether sitting for long periods was the cause or consequence of MDD. Reverse causality is recognized as a potential confounder for the observed association. Second, there could be a cyclical and reciprocal association between sitting-time and MDD because of the nature of mental health conditions, which could lead to overestimation in cross-sectional studies. Third, our research might have validity issues from using self-reported measures of PA and sitting time. In this study, sitting time was assessed by only one question, which asked about a typical day including both work and leisure time. A previous study estimated sitting time separately for weekdays and weekends, as well as for time spent sitting at work or while travelling [30]. In contrast, survey questions in our study did not distinguish between work and non-work days, or work-related or leisure-related sitting time; therefore, we were unable to tell whether specific sitting time affected MDD. However, the aim of the study was to identify the effects of overall sitting time and PA on MDD, and further research should be conducted to overcome this limitation. Fourth, we might have issues with representativeness, as we excluded unavailable missing data which accounted for nearly 20% of the original survey. Therefore, further research should be considered to make up for the missing data. Fifth, the potential health risks related to valuable evidence could be highly prevalent in contemporary society. In fact, South Korea experienced a serious tragic social event in 2014, which could affect depressed people for a long period and might have contributed to overall reductions or increases in sitting time, affecting MDD.

Nevertheless, the study is unique, because it included a representative sample of the population of an entire country and was the first to examine the relationship between sitting time and MDD in South Korea. Moreover, it assessed differences in MDD according to PA and sitting time.

## Conclusions

This study showed that sitting for long periods was associated with greater risk of MDD in South Korean adults. The findings accentuated the importance of reducing overall sitting time and increasing PA and suggested that policymakers should develop strategies involving PA, to decrease sitting time and alleviate the burden of depression in terms of fiscal health premiums and social problems.

## Additional file

Additional file 1: Table S1. Men and women participants’ general characteristics. * Number of chronic diseases: Hypertension, dyslipidemia, stroke, myocardial infarction, angina, arthritis, rheumatoid arthritis, asthma, thyroid gland disorder, chronic renal failure, hepatitis B. * Number of chronic diseases: Hypertension, dyslipidemia, stroke, myocardial infarction, angina, arthritis, rheumatoid arthritis, asthma, thyroid gland disorder, chronic renal failure, hepatitis B. **Table S2.** Subgroup analysis of sitting-time and major depressive disorder according to physical activity. Adjusted for age, household income level, educational level, marital status, occupation, obesity, current smoking status, alcohol use and number of chronic diseases. (DOCX 51 kb)

## Abbreviations

CVD: Cardiovascular disease
IPAQ: International physical activity questionnaire
KCDC: Korea centers for disease control and prevention
KNHANES: Korean national health and nutrition examination survey
MDD: Major depressive disorder
OECD: Organization for economic co-operation and development
PA: Physical activity
PHQ-9: Patient Health Questionnaire
SB: Sedentary behavior
