# Supplementary material for: The impact of sitting time and physical activity on major depressive disorder in South Korean adults: a cross-sectional study
Source: BMC Psychiatry. 2017 Jul 28;17:274. doi: 10.1186/s12888-017-1439-3 (PMC5534054; doi:10.1186/s12888-017-1439-3)
Supplement: Additional file 1: Table S1. — Men and women participants’ general characteristics. * Number of chronic diseases: Hypertension, dyslipidemia, stroke, myocardial infarction, angina, arthritis, rheumatoid arthritis, asthma, thyroid gland disorder, chronic renal failure, hepatitis B. * Number of chronic diseases: Hypertension, dyslipidemia, stroke, myocardial infarction, angina, arthritis, rheumatoid arthritis, asthma, thyroid gland disorder, chronic renal failure, hepatitis B. Table S2. Subgroup analysis of sitting-time and major depressive disorder according to physical activity. Adjusted for age, household income level, educational level, marital status, occupation, obesity, current smoking status, alcohol use and number of chronic diseases. (DOCX 51 kb) [file 12888_2017_1439_MOESM1_ESM.docx]

**Additional file 1. Men and women participants’ general characteristics**

|  |  | **Depression(men)** | | | | | | |
| --- | --- | --- | --- | --- | --- | --- | --- | --- |
|  |  | **Total** | | **Yes** | | **No** | |  |
|  |  | **N** | **(%)** | **N** | **(%)** | **N** | **(%)** | **p-value** |
| **Sitting time(hours)** | |  |  |  |  |  |  |  |
|  | <5 | 432 | (25.96) | 25 | (5.79) | 407 | (94.21) | 0.1137 |
|  | 5-7 | 454 | (27.28) | 23 | (5.07) | 431 | (94.93) |  |
|  | 8-10 | 445 | (26.74) | 34 | (7.64) | 411 | (92.36) |  |
|  | >10 | 333 | (20.01) | 30 | (9.01) | 303 | (90.99) |  |
| **Age(year)** | |  |  |  |  |  |  |  |
|  | 20-29 | 164 | (9.86) | 14 | (8.54) | 150 | (91.46) | 0.3433 |
|  | 30-39 | 290 | (17.43) | 25 | (8.62) | 265 | (91.38) |  |
|  | 40-49 | 278 | (16.71) | 17 | (6.12) | 261 | (93.88) |  |
|  | 50-59 | 308 | (18.51) | 18 | (5.84) | 290 | (94.16) |  |
|  | 60-69 | 322 | (19.35) | 15 | (4.66) | 307 | (95.34) |  |
|  | 70+ | 302 | (18.15) | 23 | (7.62) | 279 | (92.38) |  |
| **Household income level** | |  |  |  |  |  |  |  |
|  | Low | 280 | (16.83) | 42 | (15.00) | 238 | (85.00) | <.0001 |
|  | Lower middle | 396 | (23.80) | 26 | (6.57) | 370 | (93.43) |  |
|  | Upper middle | 512 | (30.77) | 22 | (4.30) | 490 | (95.70) |  |
|  | High | 476 | (28.61) | 22 | (4.62) | 454 | (95.38) |  |
| **Educational levels** | |  |  |  |  |  |  |  |
|  | Middle school or below | 495 | (29.75) | 42 | (8.48) | 453 | (91.52) | 0.0801 |
|  | High school | 561 | (33.71) | 39 | (6.95) | 522 | (93.05) |  |
|  | Above college | 608 | (36.54) | 31 | (5.10) | 577 | (94.90) |  |
| **Occupation** | |  |  |  |  |  |  |  |
|  | White collar | 463 | (27.82) | 17 | (3.67) | 446 | (96.33) | 0.0003 |
|  | Pink collar | 155 | (9.31) | 16 | (10.32) | 139 | (89.68) |  |
|  | Blue collar | 586 | (35.22) | 33 | (5.63) | 553 | (94.37) |  |
|  | Unemployed, housewife or students | 460 | (27.64) | 46 | (10.00) | 414 | (90.00) |  |
| **Marital status** | |  |  |  |  |  |  |  |
|  | Single | 274 | (16.47) | 28 | (10.22) | 246 | (89.78) | 0.0016 |
|  | Married | 1292 | (77.64) | 72 | (5.57) | 1220 | (94.43) |  |
|  | Separated | 98 | (5.89) | 12 | (12.24) | 86 | (87.76) |  |
| **Practice of aerobic exercise per week** | |  |  |  |  |  |  |  |
|  | No | 715 | (42.97) | 52 | (7.27) | 663 | (92.73) | 0.4437 |
|  | Yes | 949 | (57.03) | 60 | (6.32) | 889 | (93.68) |  |
| **Obesity (BMI, kg/m^2^)** | |  |  |  |  |  |  |  |
|  | Low weight (< 18.5) | 45 | (2.70) | 9 | (20.00) | 36 | (80.00) | 0.0011 |
|  | Normal (18.5- 24.9) | 1004 | (60.34) | 60 | (5.98) | 944 | (94.02) |  |
|  | High weight ( ≥ 25.0) | 615 | (36.96) | 43 | (6.99) | 572 | (93.01) |  |
| **Current Smoking status** | |  |  |  |  |  |  |  |
|  | No | 1025 | (61.60) | 52 | (5.07) | 973 | (94.93) | 0.0006 |
|  | Yes | 639 | (38.40) | 60 | (9.39) | 579 | (90.61) |  |
| **Alcohol use** | |  |  |  |  |  |  |  |
|  | No | 483 | (29.03) | 31 | (6.42) | 452 | (93.58) | 0.7449 |
|  | Yes | 1181 | (70.97) | 81 | (6.86) | 1100 | (93.14) |  |
| **Number of chronic diseases*** | |  |  |  |  |  |  |  |
|  | 0 | 862 | (51.80) | 53 | (6.15) | 809 | (93.85) | 0.2327 |
|  | 1 | 539 | (32.39) | 35 | (6.49) | 504 | (93.51) |  |
|  | ≥ 2 | 263 | (15.81) | 24 | (9.13) | 239 | (90.87) |  |
| **Total** |  | 1664 | (100.00) | 112 | (6.73) | 1552 | (93.27) |  |

* Number of chronic diseases: Hypertension, dyslipidemia, stroke, myocardial infarction, angina, arthritis, rheumatoid arthritis, asthma, thyroid gland disorder, chronic renal failure, hepatitis B.

**Additional file 1. Men and women participants’ general characteristics (continued)**

|  |  | **Depression(women)** | | | | | | |
| --- | --- | --- | --- | --- | --- | --- | --- | --- |
|  |  | **Total** | | **Yes** | | **No** | |  |
|  |  | **N** | **(%)** | **N** | **(%)** | **N** | **(%)** | **p-value** |
| **Sitting time(hours)** | |  |  |  |  |  |  |  |
|  | <5 | 627 | (25.27) | 61 | (9.73) | 566 | (90.27) | 0.0067 |
|  | 5-7 | 741 | (29.87) | 84 | (11.34) | 657 | (88.66) |  |
|  | 8-10 | 707 | (28.50) | 102 | (14.43) | 605 | (85.57) |  |
|  | >10 | 406 | (16.36) | 65 | (16.01) | 341 | (83.99) |  |
| **Age(year)** | |  |  |  |  |  |  |  |
|  | 20-29 | 258 | (10.40) | 39 | (15.12) | 219 | (84.88) | 0.0002 |
|  | 30-39 | 463 | (18.66) | 44 | (9.50) | 419 | (90.50) |  |
|  | 40-49 | 450 | (18.14) | 39 | (8.67) | 411 | (91.33) |  |
|  | 50-59 | 498 | (20.07) | 59 | (11.85) | 439 | (88.15) |  |
|  | 60-69 | 436 | (17.57) | 63 | (14.45) | 373 | (85.55) |  |
|  | 70+ | 376 | (15.16) | 68 | (18.09) | 308 | (81.91) |  |
| **Household income level** | |  |  |  |  |  |  |  |
|  | Low | 479 | (19.31) | 115 | (24.01) | 364 | (75.99) | <.0001 |
|  | Lower middle | 634 | (25.55) | 79 | (12.46) | 555 | (87.54) |  |
|  | Upper middle | 702 | (28.30) | 66 | (9.40) | 636 | (90.60) |  |
|  | High | 666 | (26.84) | 52 | (7.81) | 614 | (92.19) |  |
| **Educational levels** | |  |  |  |  |  |  |  |
|  | Middle school or below | 944 | (38.05) | 154 | (16.31) | 790 | (83.69) | <.0001 |
|  | High school | 771 | (31.08) | 89 | (11.54) | 682 | (88.46) |  |
|  | Above college | 766 | (30.87) | 69 | (9.01) | 697 | (90.99) |  |
| **Occupation** | |  |  |  |  |  |  |  |
|  | White collar | 464 | (18.70) | 33 | (7.11) | 431 | (92.89) | <.0001 |
|  | Pink collar | 345 | (13.91) | 37 | (10.72) | 308 | (89.28) |  |
|  | Blue collar | 403 | (16.24) | 43 | (10.67) | 360 | (89.33) |  |
|  | Unemployed, housewife or students | 1269 | (51.15) | 199 | (15.68) | 1070 | (84.32) |  |
| **Marital status** | |  |  |  |  |  |  |  |
|  | Single | 290 | (11.69) | 38 | (13.10) | 252 | (86.90) | <.0001 |
|  | Married | 1777 | (71.62) | 187 | (10.52) | 1590 | (89.48) |  |
|  | Separated | 414 | (16.69) | 87 | (21.01) | 327 | (78.99) |  |
| **Practice of aerobic exercise per week** | |  |  |  |  |  |  |  |
|  | No | 1253 | (50.50) | 176 | (14.05) | 1077 | (85.95) | 0.0256 |
|  | Yes | 1228 | (49.50) | 136 | (11.07) | 1092 | (88.93) |  |
| **Obesity (BMI, kg/m^2^)** | |  |  |  |  |  |  |  |
|  | Low weight (< 18.5) | 126 | (5.08) | 24 | (19.05) | 102 | (80.95) | 0.0711 |
|  | Normal (18.5- 24.9) | 1648 | (66.42) | 198 | (12.01) | 1450 | (87.99) |  |
|  | High weight ( ≥ 25.0) | 707 | (28.50) | 90 | (12.73) | 617 | (87.27) |  |
| **Current Smoking status** | |  |  |  |  |  |  |  |
|  | No | 2383 | (96.05) | 288 | (12.09) | 2095 | (87.91) | 0.0003 |
|  | Yes | 98 | (3.95) | 24 | (24.49) | 74 | (75.51) |  |
| **Alcohol use** | |  |  |  |  |  |  |  |
|  | No | 1503 | (60.58) | 195 | (12.97) | 1308 | (87.03) | 0.458 |
|  | Yes | 978 | (39.42) | 117 | (11.96) | 861 | (88.04) |  |
| **Number of chronic diseases*** | |  |  |  |  |  |  |  |
|  | 0 | 1377 | (55.50) | 143 | (10.38) | 1234 | (89.62) | <.0001 |
|  | 1 | 594 | (23.94) | 75 | (12.63) | 519 | (87.37) |  |
|  | ≥ 2 | 510 | (20.56) | 94 | (18.43) | 416 | (81.57) |  |
| **Total** |  | 2481 | (100.00) | 312 | (12.58) | 2169 | (87.42) |  |

* Number of chronic diseases: Hypertension, dyslipidemia, stroke, myocardial infarction, angina, arthritis, rheumatoid arthritis, asthma, thyroid gland disorder, chronic renal failure, hepatitis B.

**Additional file 2. Subgroup analysis of sitting-time and major depressive disorder according to physical activity**

|  |  |  |  | **Depression** | | | **Depression(men)** | | | **Depression (women)** | | |
| --- | --- | --- | --- | --- | --- | --- | --- | --- | --- | --- | --- | --- |
|  |  |  |  | **OR** | **95% CI** | | **OR** | **95% CI** | | **OR** | **95% CI** | |
| **Physical activity (min/week)** | | **Sitting time(hours)** | |  |  |  |  |  |  |  |  |  |
|  | No (0-149) |  | <5 | 1.00 |  |  | 1.00 |  |  | 1.00 |  |  |
|  |  |  | 5-7 | 1.13 | (0.73- | 1.75) | 1.04 | (0.35- | 3.10) | 1.16 | (0.71- | 1.89) |
|  |  |  | 8-10 | 1.78 | (1.17- | 2.70) | 3.04 | (1.15- | 8.01) | 1.56 | (0.97- | 2.51) |
|  |  |  | >10 | 1.57 | (0.98- | 2.50) | 3.43 | (1.26- | 9.35) | 1.23 | (0.71- | 2.14) |
|  |  |  | P for trend | 0.009 |  |  | 0.003 |  |  | 0.209 |  |  |
|  | Yes (≥150) |  | <5 | 1.00 |  |  | 1.00 |  |  | 1.00 |  |  |
|  |  |  | 5-7 | 1.07 | (0.69- | 1.66) | 0.93 | (0.42- | 2.06) | 1.26 | (0.73- | 2.16) |
|  |  |  | 8-10 | 1.35 | (0.86- | 2.11) | 1.03 | (0.44- | 2.40) | 1.64 | (0.94- | 2.85) |
|  |  |  | >10 | 1.94 | (1.19- | 3.15) | 1.68 | (0.71- | 4.00) | 2.27 | (1.23- | 4.21) |
|  |  |  | P for trend | 0.006 |  |  | 0.286 |  |  | 0.006 |  |  |

Adjusted for age, household income level, educational level, marital status, occupation, obesity, current smoking status, alcohol use and number of chronic diseases.
